# BK channel activators and their therapeutic perspectives

**DOI:** 10.3389/fphys.2014.00389

**Published:** 2014-10-09

**Authors:** Bo H. Bentzen, Søren-Peter Olesen, Lars C. B. Rønn, Morten Grunnet

**Affiliations:** ^1^Department of Biomedical Sciences, Faculty of Health Sciences, Danish Arrhythmia Research Centre, University of CopenhagenCopenhagen, Denmark; ^2^Acesion PharmaCopenhagen, Denmark; ^3^H. Lundbeck A/SCopenhagen, Denmark

**Keywords:** KCa1.1, BK, MaxiK, *KCNMA1*, high-conductance calcium-activated potassium channels, activators, openers, pharmacology

## Abstract

The large conductance calcium- and voltage-activated K^+^ channel (KCa1.1, BK, MaxiK) is ubiquitously expressed in the body, and holds the ability to integrate changes in intracellular calcium and membrane potential. This makes the BK channel an important negative feedback system linking increases in intracellular calcium to outward hyperpolarizing potassium currents. Consequently, the channel has many important physiological roles including regulation of smooth muscle tone, neurotransmitter release and neuronal excitability. Additionally, cardioprotective roles have been revealed in recent years. After a short introduction to the structure, function and regulation of BK channels, we review the small organic molecules activating BK channels and how these tool compounds have helped delineate the roles of BK channels in health and disease.

## Introduction

The large conductance calcium- and voltage-activated K^+^ channel (BK, KCa1.1, MaxiK) channel is encoded by a single gene (*KCNMA1*, *SLO-1*). Structurally the channel consist of four pore-forming BK α-subunits, each with 7 transmembrane segments and an extracellular N-terminus. The channel monomer is composed of essentially two distinct modules, one being primarily responsible for voltage-sensing and the other for Ca^2+^-sensing (Meera et al., 1997).

The BK channel is ubiquitously expressed in the body and represents a unique class of potassium channels, not only because of its high single channel conductance (Butler et al., 1993; Pallanck and Ganetzky, 1994) (~250 pS measured in symmetrical K^+^), but also because it can be activated by Ca^2+^ alone, membrane depolarization alone, or synergistically by both (Magleby, 2003). The ability to integrate changes in intracellular calcium and membrane potential makes the BK channel an important negative feedback system, linking changes in intracellular calcium to outward hyperpolarizing potassium currents. Consequently, the channel has many important roles, including regulation of neurotransmitter release, neuronal excitability and smooth muscle tone (Salkoff et al., 2006). In agreement with this experimental down-regulation of BK in mice is associated with erectile dysfunction, bladder over-activity, urinary incontinence (Meredith et al., 2004; Werner et al., 2005; Sprossmann et al., 2009) and hypertension (Sausbier et al., 2005). In addition, a gain-of-function mutation in *KCNMA1* has been reported to result in a syndrome of coexistent generalized epilepsy and paroxysmal dyskinesia in humans (Du et al., 2005); Gain-of-function mutations in the β1 subunit were associated with low prevalence of diastolic hypertension (Fernández-Fernández et al., 2004). However, the role of BK channels in epilepsy is not straight forward, as a loss of function mutation has also been associated with temporal lobe epilepsy, as described in detail in the paragraph “*BK channels in the central nervous system.*”

To serve its many diverse roles, and considering its wide tissue distribution, it is not surprising that BK activity is regulated by numerous mechanisms. On the transcriptional level, extensive alternative splicing gives rise to channels with altered Ca^2+^ sensitivity, channel kinetics, localization and hormone sensitivity (for review see Shipston, 2001). Moreover co-assembly with auxillary subunits including β1-4 and leucine-rich repeat-containing proteins (LRRC) changes the channels calcium- and voltage-sensitivity, as well as pharmacological properties (Knaus et al., 1994; McManus et al., 1995; Valverde et al., 1999; Wallner et al., 1999; Meera et al., 2000; Yan and Aldrich, 2010, 2012). On the post-translational level, channel activity is regulated by a number of endogenous mediators such as arachidonic acids, NO, pH (Avdonin et al., 2003), zinc (Hou et al., 2010), and phosphorylation of the channel (Yan et al., 2008) by protein kinase A, C, G, and CaMKII (Hou et al., 2009; Wang et al., 2013; Hosseinzadeh et al., 2014; Toro et al., 2014). Other modifications such as palmitoylation favor cell surface expression, while myristoylation appear to favor endocytosis (Jeffries et al., 2010; Alioua et al., 2011; Tian et al., 2012). Finally, BK channels have been found to localize to macro-molecular signaling complexes that also contain calcium channels (Grunnet and Kaufmann, 2004; Fakler and Adelman, 2008) or in close proximity to the IP3-receptors and ryanodine receptors (Nelson et al., 1995; Weaver et al., 2007). This co-localization to areas of high local calcium levels adds a regulatory level. Consequently, it is difficult to predict the activity of BK channels in a given cell; but because of their synergistic activation by voltage and calcium, one can assume that pharmacological activation of the channel will tend to potentiate the existing physiological regulatory role that BK channels play. Hence, under most physiological conditions, drugs that increase the open probability of BK channels will promote an efflux of potassium ions upon increases in intracellular Ca^2+^. This hyperpolarization of cell membranes leads to cellular responses such as decreased cell excitability and smooth muscle relaxation. Due to this, drugs that activate BK channels could represent a novel therapeutic strategy for treatment of certain types of epilepsy, bladder instability, erectile dysfunction, ischemic heart disease and chronic obstructive pulmonary disease. Despite intense academic and industrial focus, no drugs intentionally targeting BK channels are on the market and to the best of our knowledge only one drug (Andolast) is currently in clinical development.

## BK channel activators

Many different chemical entities have been found to increase the activity of BK channels. Within these entities, differences in calcium dependency, subunit composition and drug binding sites have been found. Based on their origin and structure the chemical entities can be classified in: (A) Endogenous BK channel modulators and structural analogs; (B) Naturally occurring BK channel openers and structural analogs; (C) Synthetic BK channel openers. Only a handful of interesting examples will be provided. For a comprehensive review see Nardi and Olesen (2008).

### Endogenous BK channel modulators and structural analogs

Endogenous chemicals such as arachidonic acid and the metabolites of cytochrome P450, epoxygenase and lipoxygenase, have been found to increase BK channel activity and be important regulators of vascular tone (for review see Félétou, 2009; Hou et al., 2009). Likewise, other unsaturated free fatty acids such as the omega-3 docosahexaenoic acid have also been found to increase BK channel activity (Denson et al., 2000) in an β-subunit dependent manner (Hoshi et al., 2013a). *In vivo* studies involving omega-3 docosahexaenoic acid report lowering of blood pressure in anesthetized wild-type, but not in *Slo-1* knockout mice (Hoshi et al., 2013b). Direct activation of BK channels by the sex hormone 17β-estradiol has also been reported, with its effect being dependent on the presence of the β-1 subunit (Valverde et al., 1999). 17β-estradiol was later shown to protect cardiac cells from ischemia reperfusion injuries via a possible mitochondrial BK effect, which led the authors to speculate its possible contribution to the increased incidence of post-menopausal heart attack (Ohya et al., 2005). Likewise other steroid hormones also modulate BK channel activity in a β-subunit dependent manner (King et al., 2006). Later it was found that already marketed drugs such as the xenoestrogen Tamoxifen also displays BK channel activation (Dick et al., 2001). However, the binding site and mechanism of both estrogen and xenoestrogens are still largely unknown. Other drugs on the market targeting CNS excitability and smooth muscle contraction also display BK channel activating properties in the therapeutic concentration range. These include the anti-epileptics zonisamide (Huang et al., 2007) and chlorzoxazone (Liu et al., 2003), along with the phosphodiesterase III inhibitor (cilostazol) approved for treatment of intermittent claudication (Wu et al., 2004).

Recently the first peptide BK channel activator was described. Human β-defensin 2 is an antimicrobial peptide that is important in the innate immune system. It was found to increase the open probability of the BK channel complex when applied in physiologically relevant concentrations, with the effect being dependent on two amino-acids in the β-1 loop. Interestingly the authors further demonstrated that this peptide was down-regulated in sera from hypertensive patients, and that infusion of human β-defensin 2 in monkeys significantly reduced blood pressure (Liu et al., 2013).

### Naturally occurring BK channel openers and structural analogs

Natural occurring BK channel activators are found in herbs, roots, fungi, and leaves, and have been used in folk medicine for treatment of asthma and smooth muscle disorders (Nardi et al., 2003). Isolation of natural occurring compounds revealed several BK channel activators including DHS-I that was found to dramatically increase the open probability of smooth muscle BK channels when applied to the intracellular site (McManus et al., 1993). The effect like many openers was dependent on the presence of β-1 subunit (McManus et al., 1995).

### Synthetic BK channel openers

A wealth of synthetic BK channel activators capable of increasing channel open probabilities have been synthesized by a number of companies. NeuroSearch was among the first to report on small molecule BK channel activators such as NS004 (Olesen et al., 1994a). Later, another benzimidazolone in the form of NS1619 was introduced (Olesen et al., 1994b), which turned out to be the most used tool compound for studying the functional effect of BK channels. NS1619 has been used to study the involvement and therapeutic potential in smooth muscle disorders such as pulmonary hypertension (Vang et al., 2010; Revermann et al., 2014), erectile dysfunction (Spektor et al., 2002; González-Corrochano et al., 2013; Király et al., 2013), bladder instability (Soder and Petkov, 2011; La Fuente et al., 2014) and shock-induced vascular hyperactivity (Hu et al., 2014) along with research into migraines (Lu et al., 2014), inflammatory pain (Akerman et al., 2010), and cytoprotection (Xu et al., 2002; Gáspár et al., 2009).

The binding site of NS1619 has not been established. However, a recent report on another BK channel activator, Cym04, found that the activating effects of both drugs were dependent on the S6/RCK (Regulator of Conductance for K^+^) linker; the sensitivity to both drugs was lost in BK channel variants with a distinct S6/RCK linker sequence having two deletion mutations. Whether the linker serves as a direct binding site, or is merely important for indirect conformational changes that lead to activation induced by the drug, is not known (Gessner et al., 2012). This linker has previously been proposed to act as a spring that transmits the conformational changes in the RCK upon calcium binding to opening of the channel's gate (Jiang et al., 2002).

The use of NS1619 is however hampered by a relative poor potency, and many off target effects such as inhibition of calcium channels. Consequently, we generated the biarylthiourea NS11021 as a more selective and potent tool compound to study BK channel function. Like many other activators, NS11021 works by shifting the voltage-activation curve of the channel to more negative potentials. At the single channel level this is reflected in an increased open probability (Bentzen et al., 2007). This compound has been found to reduce infarct sizes and increase cardiac performance after ischemia, to improve mitochondrial respiration and to protect isolated cardiomyocytes from reperfusion injuries (Bentzen et al., 2009; Aon et al., 2010; Borchert et al., 2013). With regard to studies on smooth muscle dysfunction, NS11021 has been found to enhance erectile responses in rats primarily through its BK activating effects (Kun et al., 2009), and to evoke a pronounced relaxation in small human penile arteries (Király et al., 2013), both equipotent to that induced by the phosphodiesterase type-5 inhibitor sildenafil. In the guinea pig bladder NS11021 decreased excitability and contractility of urinary bladder smooth muscle. This effect was antagonized by iberiotoxin, a selective toxin inhibitor of BK channels (Layne et al., 2010). In the CNS NS11021 has been used to establish a role of BK channels in migraine (Wulf-Johansson et al., 2010; Liu et al., 2014). More recently a new family of BK channel activators called the GoSlo-SR family was introduced. They are capable of shifting the voltage dependence of BK activation by more than −100 mV when applied at 10 μM to rabbit bladder smooth muscle cells (Roy et al., 2012), with newer derivatives producing the same effects at lower concentrations (Roy et al., 2014). The voltage-sensitive dye bis-(1, 3-dibutylbarbituric acid) trimethine oxonol [DiBAC4(3)] at submicromalor levels (Morimoto et al., 2007) produced a shift in the voltage-dependence of the BK channel only when β-1 and β-4 subunits were present, whereas saturating concentrations (30 μM), produced huge negative shifts by up to −300 mV in the voltage-dependent activation of BK channels, but this effect was neither dependent on calcium nor the presence of β-subunits (Scornik et al., 2013). Subunit dependent potency and efficacy was also recently demonstrated for a series of N-arylbenzamide, with loss of effect when β-1 subunits were co-expressed (Kirby et al., 2013). We recently reported a novel positive BK channel modulator, NS19504 identified in a HTS FLIPR screen (Nausch et al., 2014). This compound represent a novel chemical scaffold and was observed to activate endogenous BK channels in native smooth muscle cells from guinea pig bladder, and to reduce spontaneous contractions in bladder strips in an iberiotoxin-sensitive manner. This suggests NS19504 could serve as a tool to elucidate BK channel function in complex tissues.

Over the last decades a range of structurally distinct synthetic BK channel activators have been produced by different pharmaceutical companies. One of these was BMS204352 that showed neuroprotective effects in animal models (Cheney et al., 2001), and was advanced to phase III clinical trials for the treatment of acute ischemic stroke but failed. A related compound, BMS223131, was shown to relax smooth muscle (Boy et al., 2004) and studied clinically for overactive bladder, but not developed. Other newly synthesized small molecule BK channel activators include thioureas, tetrahydroquinolines, such as compound 36 (Gore et al., 2010) and compound Z (Ponte et al., 2012), terpenes and benzofuroindoles. These were intended mostly for the treatment of urinary incontinence, overactive bladder, erectile dysfunction and stroke, and some of the drugs entered phase I and II clinical trials (for a comprehensive review of synthetic BK channel activators please refer to Nardi and Olesen, 2008). To our knowledge the only BK channel activating drug still considered for clinical development is Andolast. It is neither selective nor potent, but according to the manufacture, Rottapharm, it has shown clinical efficacy and an acceptable safety profile in mild/moderate asthma. However, phase III clinical trials have not been commenced.

## BK channels in the working myocardium

Following a coronary artery occlusion, early restoration of blood perfusion to the ischemic myocardium by reperfusion therapy is of most importance in order to salvage the heart (Keeley et al., 2003). Paradoxically, restoration of blood flow to an ischemic myocardium also causes a so-called reperfusion injury by which cardiac myocytes that were viable immediately before reperfusion die giving rise to an increased infarct size. Because infarct size is recognized as the major determinant of myocardial functional recovery and mortality, any therapy aimed at reducing reperfusion injuries would be appreciated (for review see Yellon and Hausenloy, 2007). In 1986 Murry and colleagues were the first to demonstrate such an intervention when they showed that four short cycles of ischemia interspersed by reperfusion before the sustained ischemic event resulted in a dramatic reduction in infarct size of ~75% (Murry et al., 1986). The nature of this protective mechanism probably involves cardiac K^+^ channels.

The role of BK channels in cardiomyocytes was for many years neglected because of their absence from the plasma membrane of cardiomyocytes.

However, in 2002 the group of Brian O'Rourke demonstrated the presence of mitochondrial BK channel in cardiomyocytes (Xu et al., 2002). Patch-clamp recordings on mitoplasts from isolated guinea pig cardiomyocytes revealed a voltage- and calcium-dependent potassium current, with a single channel conductance of 307 pS that could be blocked by charybdotoxin, thereby resembling the properties of the plasma membrane BK channel, oriented with its C-terminal facing the mitochondrial matrix. They further demonstrated that mitochondrial uptake of K^+^ was blunted by charybdotoxin and iberiotoxin and accelerated by the BK channel activator NS1619. The molecular identity of the channel remained unknown, but a link to cardioprotection was made by demonstrating that NS1619 when administered prior to the ischemic event, protected isolated perfused rabbit hearts from global ischemia and reperfusion injury, and that this effect was blocked when the BK channel blocker paxilline was co-administered (Xu et al., 2002). Subsequent studies have confirmed that administration of NS1619 protects the heart from ischemia-reperfusion injury both in mice (Wang et al., 2004; Redel et al., 2008), rats (Cao et al., 2005; Gao et al., 2005) guinea pigs (Stowe et al., 2006), rabbits (Shi et al., 2007), dogs (Shintani et al., 2004), and in aged rats (Heinen et al., 2014). Likewise, we found that NS11021 protected the heart from ischemia-reperfusion injury when applied prior to, or immediately after ischemia and that this could be antagonized by paxilline (Bentzen et al., 2009).

Because most of the data addressing the role of mitochondrial BK channels rely on pharmacological tools, controversies exist about the importance of mitochondrial BK channels in cardioprotection, as it has been found that both the activators and inhibitors used display unspecific effects. NS1619 at higher concentrations directly inhibits L-type Ca^2+^ channels in rat ventricular myocytes (Park et al., 2007), Ca^2+^-activated chloride currents (Saleh et al., 2007), and voltage-activated Ca^2+^, K^+^, and Na^+^ channels (Edwards et al., 1994; Olesen et al., 1994b; Holland et al., 1996). Moreover, Cancherini et al. demonstrated that NS1619 also display non-ion channel effects on mitochondria, which they claim could explain some of the cardioprotective effects of NS1619 (Cancherini et al., 2007). A study using the cardioprotective BK channel activator NS11021, found that in nanomolar concentration NS11021 displayed beneficial effects on mitochondria, whereas when the concentration was increased, unspecific effects not related to mitochondrial BK channels were observed (Aon et al., 2010). Interestingly, a methylated analog of NS11021 that is inactive on BK channel, and does not provide cardioprotection (Bentzen et al., 2010) still possesses the unspecific effects on mitochondria of NS11021. This supports the notion that the cardioprotection mediated by NS11021 is BK channel related and not caused by the unspecific effects of the compound (Aon et al., 2010).

With regards to the BK channel inhibitors used as tool compounds for studying cardioprotection, there is concern about the use paxilline as it has been found at higher concentrations to inhibit the sarco/endoplasmatic reticulum Ca^2+^-ATPase (SERCA) (Bilmen et al., 2002). Moreover, the cardioprotective effects of the BK channel activator isoflurane were abolished by paxilline both in wild-type and in *KCNMA1^−/−^* hearts, arguing for unspecific effects of paxilline (100). In addition, this study also suggested a dispensable role for *KCNMA1* in both mitochondrial K^+^ transport and cardioprotection. Instead, using *C. elegans* as a model system they found that the related K^+^ channel *Slo-2* was required for mitochondrial K^+^ transport and cardioprotection.

Recently a study by Singh et al. helped to settle the controversies about the pharmacology and role of mitochondrial BK channels in cardioprotection, as they describe the molecular identity of the mitochondrial BK channel. They found that the mitochondrial BK channel in the heart is encoded by a splice variant (VEDEC) of the plasma membrane *KCNMA1*, and that it holds a 50-aa splice insert which they report is essential for trafficking to the mitochondria. Moreover they also demonstrated that the cardioprotection offered by NS1619 was lost in *KCNMA1* KO mice (Singh et al., 2013), cementing the involvement of BK channels in the cardioprotection conferred by NS1619.

Additionally, Soltysinska et al. recently report electrophysiological evidence for a BK-mediated current of 190 pS in mitoplasts from wild-type but not *KCNMA1*^−/−^ cardiomyocytes. Moreover, changes in reactive oxygen species (ROS) production and attenuated oxidative phosphorylation capacities in *KCNMA1*^−/−^ cardiomyocytes were observed. This suggests a mitochondrial role of *KCNMA1* encoded BK channels in fine-tuning the oxidative state of the cell (Soltysinska et al., 2014).

The discrepancy between these two studies demonstrating a role of *KCNMA1* in mitochondria and cardioprotection and the earlier work by Wojtovich on *Slo-2* where they found *KCNMA1* to be dispensable cannot be easily resolved; it should be remembered that different genetic models and experimental procedures were utilized in conducting the research. Moreover, as the authors also state, their results do neither preclude the presence of *KCNMA1* in the mitochondria nor a role for *KCNMA1* channels in other protective paradigms (Wojtovich et al., 2011).

It is difficult to imagine how the mitochondrial BK channel can at all function considering the negative inner mitochondrial membrane potential in the range of −180 mV. However, the phosphorylation state of the channel, the presence of auxillary subunits such as β-1 (Ohya et al., 2005), β-4 (Piwonska et al., 2008; Fretwell and Dickenson, 2009; Skalska et al., 2009) and perhaps LRRCs, could allow for channel activation under physiological and pathophysiological conditions, although the role of LRRCs needs to be explored further. Moreover, considering the high single channel conductance and the large driving force for K^+^ entry, a too high open probability would be detrimental for the mitochondria, as the influx of K^+^ to the matrix would depolarize the mitochondria. Therefore, the channel needs to be present in low abundance and/or with a tightly controlled open probability so that channel activation does not dissipate the proton motive force and ionic homeostasis.

How opening of mitochondrial BK channels confer cardioprotection is largely unknown, but could take place by affecting one or more of the following parameters:

1. Mitochondrial calcium accumulation: K^+^ influx leads to a partial depolarization of the inner membrane potential (Aon et al., 2010). This will reduce the driving force for calcium entry, thereby reducing calcium overload during ischemia (Sato et al., 2005; Stowe et al., 2006). Indeed Stowe et al. found that pre-treatment with the BK channel activation by NS1619 in isolated perfused guinea pig hearts attenuated mitochondrial calcium accumulation following an ischemic event (Stowe et al., 2006). Moreover Singh et al. recently demonstrated that pre-treatment with NS1619 increased the Ca^2+^ retention capacity of mitochondria, and that this effect was lost in *KCNMA1*^−/−^ mice (Singh et al., 2013).
2. ROS production: Studies on isolated mitochondria from hearts and neurons have demonstrated a reduced ROS production following stimulation with NS1619, along with the putative activator of mitochondrial BK channels, CGS7184 (Heinen et al., 2007; Kulawiak et al., 2008). This finding was confirmed in isolated heart studies (Stowe et al., 2006). Moreover, using isolated cadiomyocyte mitochondria from *KCNMA1*^−/−^ mice it was found that the absence of BK channels resulted in elevated post-anoxic ROS levels (Soltysinska et al., 2014).
3. ATP preservation: Opening the mitochondrial BK channel has been shown to improve mitochondrial energy production, likely via swelling of the mitochondrial matrix (Aon et al., 2010). Likewise, genetically engineered loss of BK channels have been found to attenuate oxidative phosphorylation capacity (Soltysinska et al., 2014).

Taken together, at reperfusion, the improved oxidative phosphorylation, reduced ROS and mitochondrial calcium levels would prevent activation of the mitochondrial permeability transition pore (MPTP), which would otherwise lead to a non-selective permeabilization of the inner mitochondrial membrane resulting in mitochondrial collapse, termination of ATP synthesis and cell death (Szabo and Zoratti, 2014).

Indeed, experiments on mitochondria isolated from the brain have demonstrated that opening of MPTP is accelerated in the presence of iberiotoxin (Cheng et al., 2008).

The recent findings using genetically engineered models have clearly demonstrated that *KCNMA1* is important for cardioprotection and mitochondrial function. However, much exploration is still needed in order to understand the physiological role of mitochondrial BK channels and how they confer cardioprotection.

From a therapeutic perspective, the recent findings are encouraging and help to clarify the role of BK channel activators in cardioprotection. Considering the importance of BK channels in controlling vascular tone, and their role in intra-cardiac neurons (Pérez et al., 2013; Wojtovich et al., 2013) it is also important to understand how, and if these extra-cardiac BK channels contribute to cardioprotection. Somewhat surprising, and to the best of our knowledge, little research has been undertaken to explore the importance of BK channel mediated flow changes in cardioprotection. Future work using tissue specific knock-down of *KCNMA1* could shed light on this interesting aspect.

## BK channels in bladder function

The BK channel α-pore forming subunit and auxillary β-1 and β-4 subunits are expressed in urinary bladder smooth muscle (UBSM) cells (Ohya et al., 2000; Ohi et al., 2001; Petkov et al., 2001; Werner et al., 2007; Chen and Petkov, 2009; Hristov et al., 2011), where BK channels have been shown to be important regulators of bladder contractility and excitability (reviewed in Petkov, 2014).

One line of studies has demonstrated that inhibition of BK channel function markedly affects bladder function. Thus, the BK channel blockers iberiotoxin and paxilline have been shown to increase contractility in detrusor smooth muscle strips from humans (Darblade et al., 2006; La Fuente et al., 2014), pigs (Buckner et al., 2002), guinea pigs (Heppner et al., 1997; Kobayashi et al., 2000; Mora and Suarez-Kurtz, 2005), rats (Uchida et al., 2005), and mice (Herrera et al., 2005) when stimulated electrically or pharmacologically.

In addition, support for a role of BK channels in bladder function has been provided by genetic mouse models. In bladder strips from BK deficient mice lacking the BK pore forming α-subunit, contractility has been found to be markedly increased in response to both cholinergic and purinergic stimulation. This is reflected in a lower frequency of electrical stimulation required to elicit a contraction (Thorneloe et al., 2005; Werner et al., 2007). *In vivo*, *KCNMA1*^−/−^ mice without functional BK-expression show a pronounced increase in voiding frequency and the occurrence of non-voiding bladder contractions. The mice have increased average bladder pressure and decreased void volume (Meredith et al., 2004; Thorneloe et al., 2005).

Mice deficient in the BK subunit β-1, the predominant accessory subunit in smooth muscle cells show moderate hypertension (Brenner et al., 2000; Plüger et al., 2000). Bladder strips from these mice showed increased amplitude and decreased frequency of phasic contraction similar to strips from WT mice treated with iberiotoxin. However, an additional response to iberiotoxin was still present in strips from KO mice (Petkov et al., 2001). Thus, studies on BK β-1 KO mice support a role of BK and specifically the β-1 subunit in modulation of smooth muscle contractility in the cardiovascular system and bladder.

Taken together, there is considerable support that inhibition or block of BK function adversely affects bladder function, increasing contractility in mice *in vivo* and in isolated bladder preparations from several species including mice, guinea pigs, and humans. This could suggest that increased BK channel activity in the bladder may counteract excessive detrusor contractions in bladder overactivity. Indeed, in pharmacological studies, positive modulation of BK channels by means of small molecules including NS-8, NS1608, and NS1619, have been shown to at varying degrees to reduce contractility of bladder strips from rats, guinea pigs, pigs (Imai et al., 2001; Malysz et al., 2004; Mora and Suarez-Kurtz, 2005), and humans (La Fuente et al., 2014). In one study, NS8 and NS1619 reduced rat bladder and aorta contractility with approximately similar potency (Malysz et al., 2004); while in another study, using NS-8, a clear selectivity for rat bladder over portal vein was reported. This supports the notion of BK channels being an attractive target in overactive bladder and urgency urinary incontinence. NS-8 was also reported to increase bladder capacity in urethane-anaesthetized rats after duodenal administration (Nicot et al., 1992).

In detrusor myocytes, BK channels may be activated both by increases in internal Ca^2+^ mediated via voltage-dependent L-type calcium channels and transiently by ryanodine receptors mediating local “Ca^2+^ sparks” as shown by patch clamp in guinea pig and human myocytes (Ohi et al., 2001; Herrera and Nelson, 2002; Hristov et al., 2011; Malysz et al., 2013). BK channels are thereby thought to mediate a negative feedback to limit contractions of the detrusor smooth muscle (Herrera et al., 2000, 2005) and to regulate patterns of spontaneous phasic contractions. Thus, in human UBSM cells BK channel outward currents have been shown to be a major determinant of repolarization following action potentials. Moreover, BK channel activity is also an important regulator of the resting potential and spontaneous phasic contraction amplitude, as well as the amplitude of electric field stimulation-induced contractions in UBSM strips (Hristov et al., 2011).

Spontaneous non-voiding contractions have been associated with bladder overactivity (Brading, 1997; Andersson, 2010); Inhibition of spontaneous phasic contractions, with minimal effect on voiding contractions have been suggested as a favorable profile in myogenic bladder overactivity. Interestingly, decreased BK channel α- and β-subunit expression have been reported in tissue from prostate patient bladders with detrusor overactivity, when compared with patients with similar obstruction but without detrusor overactivity (Chang et al., 2010). Therefore, positive modulation of BK channels may represent a strategy to alleviate bladder overactivity and increased detrusor contractility. The BK channel modulators NS11021 and NS19504 have been studied in guinea pig strips, and shown to potently inhibit spontaneous phasic contractions with more modest effects on contractions induced by electric field stimulation (Layne et al., 2010; Nausch et al., 2014). This supports the potential of BK openers to normalize detrusor function in bladder overactivity with minimal effect on voiding contractions.

BK channel openers have also been suggested as a strategy to alleviate bladder overactivity of neurogenic origin. Interestingly, decreased BK UBSM expression, increased contractility and increased excitability in cells from humans with neurogenic detrusor overactivity have been reported (Hristov et al., 2013). However, in a study of the BK opener NS1619, effect on spontaneous contractions was only observed in detrusor strips from normal subjects, not in strips from patients with neurogenic bladder overactivity (Oger et al., 2011). In another study using isolated human UBSM cells and strips from subjects without prior history of overactive bladder, NS1619 was found to inhibit myogenic and nerve-evoked contractions (Hristov et al., 2012).

The underlying pathophysiology in overactive bladder is often not well understood and may be multifactorial, depending on both myogenic and neurogenic factors (Hanna-Mitchell et al., 2013). In addition to being a major regulator of UBSM contractility and excitability, BK channels may also be important for bladder afferent nerve activity and urothelial function. However, the role of BK channels in specific pathophysiologies associated with bladder overactivity are not known. Regulation of BK channel activity is complex; in the bladder it is regulated by voltage levels, calcium levels, kinase activity and by the action of signal molecules involved in dysregulation of UBSM function (Hristov et al., 2014). It was recently reported that BK channel activity mediates PGE2 induced increase in spontaneous phasic contractions in detrusor strips (Parajuli et al., 2014a). BK channel activity has also been linked functionally to changes in Ca^2+^ signaling and detrusor contractility induced by cAMP (Xin et al., 2014) and muscarinergic receptor activation (Parajuli et al., 2014b).

BK channel openers have been advanced to clinical studies for bladder dysfunction, but have so far not reached the market. TA-1702 (structure not disclosed) has been in Phase I by GSK under license from Tanabe. Although no public announcements have been made about withdrawal, it does not currently appear in the company's development pipeline. Nippon-Shinyaku discontinued development of NS-8 for overactive bladder (Announced Jan 16, 2007) due to lack of efficacy in proof-of-concept study with Apogepha. NS-8 reached Phase II in Europe and Phase I in Japan. A number of new BK openers representing new chemical scaffolds and showing improved selectivity have been reported, and may be promising leads for future development within bladder dysfunction.

Taken together, there is strong support that positive modulators of BK may have beneficial effect in instable or overactive bladder, without compromising normal voiding functions. Further studies using recently discovered BK openers, that possess improved selectivity are needed to translate findings from animal models to the human bladder, and to elucidate the potential for BK modulators in bladder overactivity disorders of myogenic or neurogenic origin.

## BK channels in the central nervous system

BK channels are abundantly expressed in the central nervous system (CNS) with various functions being associated to these channels in neurons. The channels are expressed in specific neurons, with subcellular localizations in presynaptic terminals, soma and dendrites (Knaus et al., 1996). As mentioned previously BK channels are distinctive among ion channels, being gated by both voltage and intracellular Ca^2+^. This gating mechanism, in combination with their close proximity or even physical coupling to voltage-gated Ca^2+^ channels, makes BK channels potentially important components in negative feed-back mechanisms (Marrion and Tavalin, 1998; Grunnet and Kaufmann, 2004). In a situation of excessive Ca^2+^ influx e.g., in pre-synaptic terminals and corresponding disproportionate transmitter release, activation of BK channels by incoming Ca^2+^ will counterbalance this effect by hyperpolarizing the membrane, and thereby shut off voltage-dependent Ca^2+^ influx. In other words, BK channels can be seen as an “emergency break,” which prevents transmitter related hyperexcitability and concomitant cell toxicity. An example of this is observed in Purkinje cells in the cerebellum (Swensen and Bean, 2003; Womack and Khodakhah, 2004). From the somatic localization, BK channels are thought to exert their function by shaping the repolarization and thereby after-hyperpolarization (AHP) of action potentials. The after-hyperpolarization is divided into three phases; a fast (fAHP), an intermediate (mAHP) and a slow (sAHP) part. BK channels participate in the fast phase and have been especially well studied in the CA1 region of the hippocampus, where fAHP is inhibited by addition of TEA and the more selective BK inhibitor iberiotoxin (Storm, 1987). It should be mentioned that BK α-subunit knock-out mice are viable, but have several phenotypes that include CNS related functions. Among these are ataxia and high frequency hearing loss (Salkoff et al., 2006).

The localization of BK channels in Purkinje cells from the cerebellum points to the important role of BK in motor coordination, since Purkinje neurons are the sole output of the cerebellar cortex. As a consequence *KCNMA1* (−/−) knock-out mice have impaired motor coordination and ataxia (Sausbier et al., 2004). These results have later been consolidated in transgenic mice lacking BK channels exclusively in the Purkinje cells of the cerebellum (Chen et al., 2010). BK channel impact on locomotor impairments may also rely on the channel expression in basal ganglia, where prominent expression of BK channels has been demonstrated in substantia nigra, pars reticulate, globus pallidus, and entopeduncular nucleus. All these areas have a potential impact on the pathophysiology of tremors and ataxia (Sausbier et al., 2006).

An auditory phenotype of the BK knock-outs also points to a function of the channels in hearing. When knocking out the BK α-subunit, but not the β-subunit, a resulting progressive hearing loss is observed. Hearing loss at high frequencies is evident, but only after 8 weeks of age and onward. This point to irreversible progressive loss of cochlear outer hair cells. A similar phenotype can be observed by genetic deletion or pharmacological inhibition of the voltage-dependent potassium channel KCNQ4 (Jentsch, 2000; Rüttiger et al., 2004).

BK channels have also been associated to circadian rhythms. Circadian rhythms in mammals are driven by a central oscillator in the hypothalamic suprachiasmatic nucleus (SCN). This nucleus is constituted by relatively few (about 10,000) neurons that are characterized to oscillate in a 24 h rhythms. These cells are unique in the sense that they can generate rhythms. The SCN neurons are central in understanding the pathophysiology of sleep and circadian diseases; these neurons are innervated from the retina and this projection is essential in setting the clock to the external light-dark regimen (entrainment). Entrainment maintains the internal rhythms of exactly 24 h.

BK channels have been suggested as a novel and attractive target for sleep and circadian diseases (Meredith et al., 2006). BK channels are expressed in the SCN, and the expression of *KCNMA1* (BK α-subunit) mRNA is regulated in a diurnal manner. Most interestingly, *KCNMA1* deficient mice have a larger amplitude in electrical activity of SCN neurons. The same mice had a weak rhythm in locomotor behavior. Taken together, these data suggest that BK modulators could influence the endogenous rhythm structure, and perhaps be useful in patients with circadian and sleep dysfunctions.

BK channels have also been associated to various epileptogenic phenotypes but the picture is not straight forward. In its simplest term, inhibition of BK channels should result in general depolarization and thereby hyperexcitability. Consequently it could be expected that epilepsy could be treated with pharmacological activation of BK channels. There is evidence of BK loss-of-function in relation to epilepsy, however to tamper the picture also BK gain-of-function has been linked to the disease. In preclinical models of inherited generalized tonic-clonic epilepsy and temporal lobe epilepsy (TLE) animals demonstrated a reduction in AHP. This decrease was suggested to be due to a reduction in Ca^2+^-dependent K^+^ conductance, that could be related to BK channels, but the exact nature of the specific conductance was not addressed by application of specific pharmacological tools (Verma-Ahuja et al., 1995). Also in a pharmacological TLE model, application of the muscarinic receptor agonist pilocarpine produced a down regulation of BK channels, especially in the hippocampus and cortex (Pacheco Otalora et al., 2008). The picture is blurred by the fact that BK channel gain-of-function, at least as a consequence of β4-subunit knock-out, that leads to an overall increase in BK activity, can result in TLE (Brenner et al., 2005). Reports in favor of increased BK activity as a cause for epilepsy, are observations that gain-of-function BK channels can result in generalized tonic-clonic seizures (Shruti et al., 2008). Target engagement in these findings was substantiated by application of paxilline that could suppress the epileptogenic phenotype (Sheehan et al., 2009).

When it comes to clinical evidence of BK channel functions, CNS is actually one of the only areas where reports exist. The first associations between BK activity and epilepsy in patients were concomitant, with the general notion of loss of BK channel activity as the underlying cause of hyperactivity and epilepsy. In TLE patients a reduction in fAHP and a down-regulation of BK channels was observed (N'Gouemo, 2011). Again the picture is distorted by the fact that human absence epilepsy and idiopathic generalized epilepsy are directly associated to a BK channel gain-of-function mutation (Du et al., 2005). The gain-of-function is a single amino acid substitution (D434G) in the BK α-subunit, that among other things, increases Ca^2+^ sensitivity and mean open time of the channel (Du et al., 2005). These observations are good examples of how the prediction of the physiological outcome of a certain ion channel mutation can be very difficult. The intuitive feeling will be that increasing a potassium conductance and thereby hyperpolarizing a cell membrane should dampen cellular excitability. However, in intact cell system this logic rational is challenged by the fact that hyperpolarization will result in more efficient release of Na^+^ channels from inactivation, resulting in a higher number of Na^+^ channels being available for activation in a subsequent action potential. The exact phenotype of a given ion channel mutation, will therefore always be the sum of the orchestrated activity of a number of different ion channels, in combination with other membrane proteins and the entire cellular machinery. When considering CNS it should also be kept in mind that the brain consists of both excitable and inhibitory neurons, and the exact interplay between neuronal circuits and cellular and subcellular positions of the mutated proteins will impact the final phenotype of any given mutation.

## Concluding remarks

It is now two decades since the first small molecule BK channel opener was reported. Over these years a wealth of BK channel openers, including naturally occurring and synthetic compounds have been discovered, and recently the first peptide opener of BK channels was disclosed (Liu et al., 2013). The structural diversity of these molecules is also reflected in their mode of action. Some compounds display β-subunit dependency (Valverde et al., 1999), whereas others are dependent upon intracellular calcium concentrations (Bentzen et al., 2007).

The pharmaceutical interest in BK channel activators has been spurred on by the last decade's biological research, reporting the important roles of BK channels in both health and disease. By integrating changes in intracellular calcium and membrane potential, the BK channel serves as an important negative feedback system, linking increases in intracellular calcium to outward hyperpolarizing potassium current. Using BK channel openers, evidence of the channels role and possible therapeutic potential has been established with respect to a number of indications, characterized by hyper excitability and smooth muscle dysfunction. Despite vast amounts of preclinical work and basic research, clinical evidence for the usefulness of BK channel openers is still missing and clinical development of BK channel activators has in large been discontinued and is to our knowledge currently not taking place except for Andolast. Translating preclinical findings to the clinic is tough, with failure being caused by many factors. However, the lack of potency and especially the poor selectivity of compounds toward off-targets are troublesome, as it can obscure preclinical proof-of-concept findings. Despite substantial amount of *in vitro* based BK pharmacology literature, it is striking how few *in vivo* evaluations have been published. Although only speculative, this could point to the challenges for *in vivo* applications of many BK activators, thereby also adding to the lack of successful clinical attempts to reveal the potential for BK activators. Therefore, a need for more potent, selective and *in vivo* applicable compounds is still warranted.

## Author contributions

All authors contributed to the drafting, writing, revising, and approval of the manuscript.

### Conflict of interest statement

The authors declare that the research was conducted in the absence of any commercial or financial relationships that could be construed as a potential conflict of interest.
